# Effects of a Cognitive Stimulation Program in Institutionalized Patients with Dementia

**DOI:** 10.3390/jpm12111808

**Published:** 2022-11-01

**Authors:** María Jiménez-Palomares, María Victoria González-López-Arza, Elisa María Garrido-Ardila, Jesús Montanero-Fernández, Trinidad Rodríguez-Domínguez, Juan Rodríguez-Mansilla

**Affiliations:** 1 ADOLOR Research Group, Department of Medical-Surgical Therapy, Medicine and Health Sciences Faculty, Extremadura University, 06006 Badajoz, Spain; 2 Mathematics Department, Medicine and Health Sciences Faculty, Extremadura University, 06006 Badajoz, Spain; 3 ROBOLAB Research Group, Medical-Surgical Therapy Department, Nursing and Occupational Therapy Faculty, Extremadura University, 10003 Cáceres, Spain

**Keywords:** major neurocognitive disorder, dementia, occupational therapy, cognitive stimulation

## Abstract

Background: The advances achieved by the available research that focus on understanding memory operation and cognitive functions have helped the development of specific treatment approaches. These can help to maintain or improve the cognitive function and well-being of people with dementia. The use of cognitive stimulation in dementia has a long history. There are multiple studies that have demonstrated its benefits on the cognitive levels of patients with mild to moderate dementia. However, all of the studies on this type of non-pharmacological intervention conclude that there is a need for more clinical trials in order to give more solidity to the evidence already found. The objective of this pilot study was to assess the effects of an occupational therapy cognitive training program on the cognitive function of institutionalized older adults with dementia. Methods: The study was a pilot randomized clinical controlled trial. A total of 58 participants with major neurocognitive disorder or dementia were randomized to the occupational therapy cognitive training program group or to the conventional occupational therapy group twice a week for 5 weeks. The cognitive level was measured with the Global Deterioration Scale (GDS) and the Lobo’s Cognitive Mini Test (LCMT), which is the Mini-Mental Status Examination in Spanish. Measures were taken at baseline (week 0), after 5 weeks of treatment (week 5), and after 6 weeks of follow up (week 12). A value of *p* < 0.05 was considered as statistically significant. Results: There were no statistical differences between groups in the LCMT global scores at baseline or after the intervention at week 5. However, the analysis of the specific cognitive areas assessed in the Lobo’s Cognitive Mini Test indicated that that the intervention group significantly improved comprehension of verbal commands and praxis (*p* = 0.021). At the follow-up measure, the differences obtained in relation to verbal commands and praxis maintained the statistical differences significantly (*p* = 0.009). Conclusions: Occupational therapy based on cognitive training shows positive effects on the maintenance of the global cognitive state of institutionalized older adults with dementia and improves significantly the comprehension of verbal commands and praxis.

## 1. Introduction

Dementia, also acknowledged as major neurocognitive disorder [1,2], is one of the main causes for the need of residential care in older adults. The estimations indicate that approximately 46 million people in the world have this condition [3,4]. In general, the most common symptoms of all types of dementia are those affecting the cognitive-intellectual, psychic, and functional spheres [2,5].

Advances in the understanding of the mechanisms of memory and the cognitive functions have led to the development of more specific treatment approaches for the management of dementia [6]. The objective of these therapies is to help maintain or improve cognitive functioning, and therefore, well-being in people with this condition [6]. Scientific evidence suggests that the rehabilitation of cognitive function is biologically possible thanks to the concept of neuroplasticity and the neuronal regenerative ability [7,8]. Currently, the importance of early detection and intervention, as well as cognitive training and rehabilitation in the management of dementia, is becoming increasingly noticeable [6]. Furthermore, the scientific evidence indicates that cognitive impairment can lead to a major loss of function in people with this condition and to an overburdening of their main caregivers [7,9].

The aim of cognitive stimulation is to increase cognitive reserve through a specific training that can restore intellectual abilities. This improvement will lead to a decrease of cognitive deterioration and will delay the negative effects that cognitive impairments have on the function of a person in their daily life activities [10]. Authors such as Clare and Wood differentiate between three types of cognitive interventions. The first is cognitive training with standard tasks in order to develop cognitive function, such as memory, attention, or executive function. The basis of this type of therapy is that the practice has the potential to improve, or at least maintain, functioning in a particular cognitive domain. The second is cognitive rehabilitation with an individualized approach to treat cognitive deficits. The aim of this approach is not to improve performance in cognitive tasks as such, but to improve functioning in the everyday context, and finally, the third type is cognitive stimulation, which includes cognitive activities which are designed to improve and preserve social and cognitive activity [11,12].

There are many studies that have carried out rehabilitation or cognitive stimulation programs in the field of dementia in our country for decades [13,14,15,16,17]. They have based their treatment on rehabilitative and therapeutic approaches. Most of them are holistic and comprehensive interventions that focus on the functional, cognitive, psycho-affective, and relational domains [7,18,19]. Several methods that are being used have a long history and were pioneers in Spain. The Integral Psychostimulation Programme (PPI) by Tarraga et al. [20], the ‘Activate the mind’ program by Peña-Casanova [21], ‘The trunk of memories’ by the Associations of Relatives of Alzheimer’s and other Dementia Patients [22] are examples of some of these programs. The use of new technologies and computers has also been integrated into treatments and there are several programs that have been developed in recent years, such as Smartbrain [23] or Gradior [13].

Occupational therapy (OT) is a profession framed within the health sciences and society. It uses meaningful or purposeful activity (occupation) which is directed and programed, as a therapeutic tool with a preventive or therapeutic purpose [24]. Occupational therapy in dementia focuses on the use of occupation to delay cognitive impairment and enhance residual abilities in order to achieve the highest possible degree of autonomy for the patient, and a better quality of life for both the patient and his or her family [19].

The role of the occupational therapist as a professional providing cognitive stimulation is supported by the study of Salmon et al. [25]. These authors indicate that cognitive stimulation is relevant to occupational therapy as it is based on important foundations of the discipline, and it focuses on the patient and on the analysis of the activity [26].

Comprehensive cognitive stimulation seems to be the way forward in the treatment approach of dementia. This type of non-pharmacological therapy has shown to be mainly effective in the cognitive field, but less so in the functional, behavioral, and affective fields [27]. The latest systematic reviews [19,28,29] advocate further research on this type of therapy in order to provide further evidence of its effects, hence the importance of this research study.

Dementia is a public health priority, and therefore needs to continue to be comprehensively studied in order to achieve an appropriate management of the condition with better preventive, diagnostic, therapeutic, and social strategies at present and in the near future [30]. Currently there is still a need for trials that focus on the direct comparison of cognitive training versus alternative non-passive treatments [28].

Therefore, the objective of this study was to assess the effects of an occupational therapy program based on cognitive training on the cognitive functioning of institutionalized older adults with dementia.

## 2. Material and Methods

### 2.1. Study Design

This pilot study was a double-blind randomized clinical controlled trial. The study was conducted and reported following the CONSORT statements [31,32] and received the ethical approval from the Bioethical Commission of the University of Extremadura in Spain (Registration number: 46/2012). The legal guardians of the participants signed a written informed consent before the commencement of the study. The trial was registered with the ClinicalTrials.gov registry (Study Identifier: NCT02199353).

### 2.2. Participants

The subjects of the study were older adults with dementia. After a three-month period of recruitment, a total of fifty eight subjects participated in the study. They were all institutionalized in ‘CARE’ residential homes (Extremadura, Spain), which are residential care centers for older adults and people in a situation of dependency. The centers provide board, lodging, and individualized care, which can include 24-h medical care and assistance of daily living depending on the needs of the person.

The established inclusion criteria were: patients with the diagnosis of dementia according to criteria of the Diagnostic and Statistical Manual of Mental Disorders (DMS IV) [33] given by a geriatrician; patients diagnosed for at least one year; over 60 years old; patients institutionalized in the residential care center for at least 6 months before the commencement of the study; patients having scores of ≤40 points in the Barthel Index [34]; patients having scores of ≤15 points in the Lobo’s Cognitive Mini Test (Mini-Mental Status Examination Spanish version) [35]; patients who were following, for at least 6 months, the occupational therapy treatment provided in the residential facilities and were also participating in the basic activities of daily living (BADL) program; and patients whose legal guardian had signed the informed consent.

The following exclusion criteria were established: patients with psychological or behavior symptoms diagnosed by a physician and Barthel Index scores under 40 points.

### 2.3. Procedure and Interventions

The participants were randomly allocated to an intervention group or a control group. The Quick Cales application (GraphPad Software, San Diego, CA, USA) allocated the ratio of participants into block sizes of three groups through a random generator used by the computer. An independent investigator who was not related to the study was responsible for the randomization procedure and the randomization list.

The control group received conventional occupational therapy in treatment room 1. This consisted of a program that combined group activities related to orientation in time and space, social activities (crafts and leisure workshops), reminiscence, and communication-building activities (talks and discussions).

The intervention group followed a cognitive occupational therapy program, which was based on the cognitive functions involved for the fulfillment of basic activities of daily life (BADL) in treatment room 2. The performance of any daily task involves multiple cognitive and motor processes. Therefore, the use of these tasks for the improvement of cognitive functions may be an appropriate approach as every basic activity of daily life has cognitive requirements [36].

The activities, the subject, the cognitive functions, and the BADL that were trained in the sessions varied, but they were always within the planning specified in the program created by the researchers. The sequence of the sessions was always the same. Every treatment session was initiated with a temporal and space orientation activity, it continued with the specific activity of the session (which corresponded to the cognitive function to be trained in that session), and was finished with a reminiscence activity. The activities were displayed on a sheet that showed the cognitive area and the BADL to be trained and included the guidelines to complete the activity. If a participant needed further clarification, the occupational therapist provided the necessary explanations to the patients to facilitate understanding and subsequent implementation of the activity. The intervention areas were selected by the researchers, according to those cognitive functions that deteriorate in dementia and are required to carry out ADL. The structure of the program and the cognitive functions that were trained during the program were included in the treatment, taking into consideration the different clinical guidelines and cognitive stimulation programs available in the literature [18,37]. These cognitive functions were the following: temporal and space orientation, memory, attention and concentration, executive function, verbal and written language, praxis, gnosis, association, and categorization. Orientation and attention were trained in all sessions as they are of prime importance for the performance of any basic ADL. The weekly planning of the treatment program can be seen in Supplemental Table S1.

An example of a session of the program can be seen in Supplemental Table S2. The cognitive function trained, the activities that were practiced, and their objectives are explained in detail.

The study was conducted during 12 weeks. The participants in the intervention and the control groups received a total of 10 treatment sessions, which were conducted twice a week for a period of 5 weeks. The treatments were carried out in individual sessions of 45 min. A follow-up assessment was carried out at week 12 after the rest period.

The patients and their caregivers were blinded as they were not aware of the study group that they were allocated to. Due to the nature of the sessions, they could identify that the patients were following the occupational therapy treatment offered in the residential home, but they were not aware of the specific treatment approach that they were receiving. In addition, the participants did not know if they were in the intervention or in the control group due to their condition and the associated symptoms.

### 2.4. Outcome Measures and Data Collection

The participants completed three assessment sessions. The first measurement was conducted at the beginning of the study and previous to the randomization (baseline or week 0), the second one was carried out once the treatment was completed (post intervention—week 5), and the last one was completed after a follow up period of 5 weeks (follow up—week 12). The assessor was an occupational therapist blinded to group allocation at all times. She was familiar with the use of the scales and was independent to the study, not being aware of the therapy applied or the objective of the study.

The following data were collected: socio-demographic data, type of dementia, concomitant treatment, and the cognitive level (main outcome measure). In order to assess the cognitive level, the following scales were used:

The Global Deterioration Scale (GDS) [38]. This scale assesses the level of cognitive deficits. The scores obtained in this scale range from 0 to 35. The results indicate: GDS 1—no cognitive deficit (30–35), GDS 2—very mild cognitive deficit (25–30), GDS 3—mild cognitive deficit (20–27), moderate cognitive deficit (16–23), moderate to severe cognitive deficit (10–19), severe cognitive deficit (0–12), and very severe cognitive deficit (0).

The Lobo’s Cognitive Mini Test (LCMT) is the Spanish version of the Mini-Mental State Examination adapted and validated by Lobo et al. in 1978 in Spain [35]. This screening test measures the cognitive level and its total score can be rated from 0 to 35 points. The interpretation of the scores obtained are as follows: 30–35 points indicate no dementia, 29–25 points indicate borderline, less than 24 points indicate the presence of dementia, 24–20 points indicate mild dementia, 19–15 points indicate moderate dementia, and 14 points or less indicate severe dementia. The MMSE is widely used worldwide, it has been translated into more than 50 languages, and is used by most specialists in geriatric medicine [39].

### 2.5. Statistical Analysis

No formal power calculation was performed as the study depended on the availability of the patients of the residential care centers. The previous studies conducted by our research group and the statistical guidelines [40] suggested that the statistical analysis to be used in the study could be justified by the recruitment of a minimum of 25 participants per group. Drop-outs were not factored into the predetermined sample size of 25 in each treatment group.

A descriptive analysis of the characteristics of the sample and the baseline measurements of the 58 participants was done, showing the distribution of categorical variables and centralization measures and the statistical dispersion and position of the continuous variables. The Kolmogorov–Smirnov test was used to analyze the normality of the continuous variables. The Pearson χ^2^ test and Fisher’s exact test (when at least 20% of the boxes in the contingency table showed a frequency of less than 5) were applied in order to assess the differences regarding the characteristics of the subjects of the intervention group and the control group at baseline. Moreover, the t-Student test and the Mann–Whitney U test were used for normal continuous variables and for non-parametric variables, respectively. The cognitive level and the cognitive function of the control group versus the intervention group at week 5 and 12 were also assessed thought the t-Student, the Pearson χ^2^, and the ratio of verisimilitude tests. The statistical analysis was performed with the SPSS Statistical software (SPSS Inc., Chicago, IL, USA), 21 version for Windows. A value of *p* < 0.05 was considered statistically significant.

In addition, a repeated measures model was applied with the Greenhouse–Geisser correction, multiplying the corresponding *p*-value by the number of dimensions analyzed in the Lobo’s Cognitive Mini Test, which were six (Bonferroni correction). Subsequently, a post hoc comparison was made between the two groups in each phase, multiplying the corresponding *p*-value by the number of phases (three).

The Little’s test was applied to corroborate if the drop-outs were missing completely at random (MCAR). An analysis of variance (ANOVA) was performed, when the variables were continuous and had normality, to analyze if the cognitive level of the intervention and the control groups had a relation to the level of cognitive deterioration (GDS).

## 3. Results

**Figure 1:**
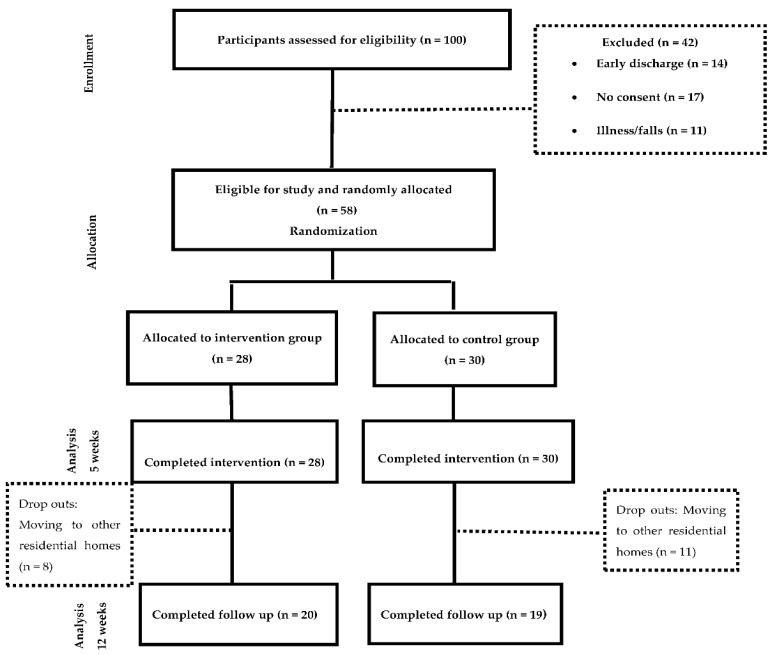
CONSORT flow diagram of study participation.

Out of the fifty-eight residents that were included in the study, 48.3% were assigned to the intervention group and 51.7% to the control group. All the participants completed the assessments at week 0 and 5. Thirty nine participants attended the follow up measurement (week 12). The drop outs were caused by the relocation of the participants to other residential care facilities. The study participation is detailed in the CONSORT flow diagram (Figure 1).

Table 1 shows the baseline socio-demographic data. The main characteristics of the intervention and the control groups at baseline were homogeneous as the Chi Square test showed. This can be observed in Table 2.

The baseline mean score in the Lobo’s Cognitive Mini Test was 21.14 for the intervention group and 21.63 for the control group (Table 3). The statistical analysis showed that, after the intervention and in the follow-up assessment, there were no statistically significant differences in the cognitive level between the two groups. Therefore, the treatment program applied to the intervention group did not produce a significant increase in the overall score of the scale.

**Figure 2:**
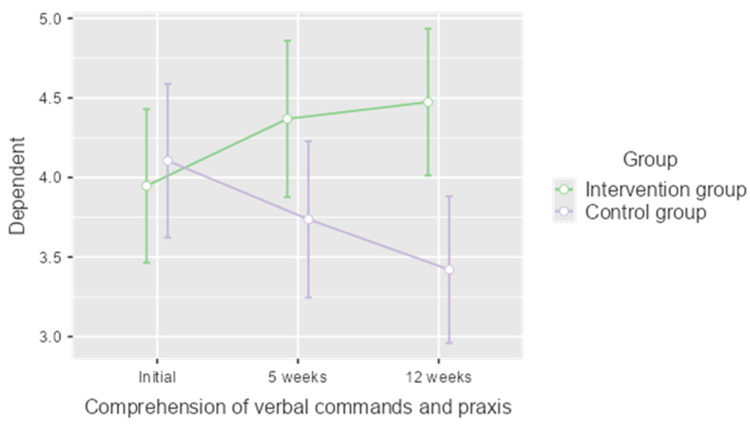
Results of the analysis of the comprehension of verbal commands and praxis dimension of the Lobo Mini Cognitive Test.

Although no significant differences were found in the general cognitive level of both groups after the treatment, an additional analysis was carried out to assess whether some of the cognitive functions assessed in the Lobo Mini Cognitive Test underwent any change. As a result of this analysis, we found that in relation to the comprehension of verbal commands and praxis dimension (Figure 2), both groups started from similar values but followed different evolutions (*p* = 0.042), so that the intervention group obtained statistically significant improvements at week 5 (*p* = 0.021) and these improvements were maintained at follow-up (*p* = 0.009).

The results of the ANOVA showed statistically significant differences between the scores of the Lobo’s Cognitive Mini Test and the GDS (Global Deterioration Scale). The related data is shown in Table 4.

The figures indicated that the cognitive level worsened significantly as the degree of dementia increased in both the control group (*p* < 0.001) and the intervention group (*p* < 0.001). This suggests that, regardless of the treatment that the participants followed, the higher the degree of dementia, the greater the cognitive impairment.

The results of the Bonferroni correction showed no statistically significant differences between groups regarding the analysis of the characteristics of the losses and the participants that completed in the study at week 0 and 5. In addition, the data obtained from Little’s test indicated that the drop-outs were missing completely at random

## 4. Discussion

This study shows that the occupational therapy program based on the cognitive training has a positive influence on the cognitive level of the patients, as the score of the Lobo’s Cognitive Mini Test increased in the intervention group after completing the treatment from 21.14 points to 23.11 points. However, this increase did not reach statistical significance in the overall Lobo’s Cognitive Mini Test score. Therefore, these results suggest that the program maintained the cognitive level of the participants without causing a decrease in the cognitive functions.

The increase experienced in the Lobo’s Cognitive Mini Test score coincides with the results of the latest reviews on the subject [28,29]. In contrast to our results, the systematic review conducted by Woods et al. in 2012 [29] concluded that after the application of stimulation programs there is a statistically significant improvement in global cognition. However, our results are closer to those obtained in the most recent reviews available and published in 2019 by Bahar-Fuchs et al. [28], which only reported small improvements.

If we focus on dementias associated with pathologies, such as Parkinson’s disease, we coincide with Orgeta et al. [41], who found that cognitive training was related with higher values of global cognition after their intervention, but did not achieve statistically significant improvements, as we did in our study.

The progressive nature of dementia implies that maintaining function for a period of time can be considered as an important achievement [7]. Therefore, our results can be considered to be relevant as the treatment increased and maintained the Lobo’s Cognitive Mini Test score for 5 weeks during the treatment without dropping back to the baseline score after 12 weeks. Consequently, we can affirm that cognitive functions were maintained throughout the duration of the research and also in the follow-up phase [28].

Apraxia is the inability to produce a correct movement in response to a verbal command, to imitate an action performed by the examiner, and to perform the movement correctly in response to a seen object [42]. Furthermore, apraxia is described as one of the domains susceptible to alteration in dementia [1].

Attention, memory, and praxis, among other neuropsychological processes, can be stimulated and enhanced by cognitive stimulation techniques [43,44]. Within the Lobo Mini Cognitive Test the dimension “comprehension of verbal commands and praxis” assesses the patient’s ability to attend, understand, and execute a complex task in three steps, copy a picture, or write a sentence. We have seen how our intervention program has led to a statistically significant improvement in this dimension (*p* = 0.021), which has continued to be maintained in the follow-up phase (*p* = 0.009). These results represent a small step forward with respect to the existing cognitive programs in which the functions that have seen improvements have mainly been memory, verbal fluency, and executive functions [28,41]. We consider that these findings open up the possibility of new approaches to cognitive stimulation.

Considering that apraxia causes difficulties in using tools and making and understanding gestures, the impairment of this cognitive function influences the activities of daily living (ADLs) of patients with dementia [45]. This interrelation would be in line with our result, as the use of cognitive activities related to ADLs has improved praxis in patients with dementia.

The study of apraxia in patients with dementia is not only important for diagnosis, but also has implications that may affect the quality of life of both patients and their caregivers [45]. Therefore, cognitive programs that produce an improvement in apraxia will result in an improvement in quality of life.

Our results are difficult to compare with other studies because the control group also received occupational therapy treatment, which included cognitive group activities. We believe that this has contributed to the fact that there is a maintenance of the cognitive level but no significant improvement. Most of the studies that apply cognitive stimulation have a control group that either does not receive therapy or is placed on a waiting list and only a few studies carry out a treatment program with this group [29]. Our study design, which involves the comparison of the intervention group with an active control group, would be supported by one of the latest reviews that analyzed 33 studies with a total of 2000 patients [28]. Their authors concluded that there is a need for clinical trials comparing cognitive stimulation with alternative treatments, rather than passive control groups [28].

A key area to be considered in studies that carry out cognitive stimulation programs is the duration and frequency of the treatment provided, as it can result in different amounts of cognitive stimulation performed [29]. In this sense, the different studies consulted in the literature range from a minimum of 5 weeks [46,47] to a maximum of 24 weeks [48], and there are interventions that even last for a year [48]. Many of the authors specify the number of sessions performed per week being between one [49,50] and five weekly sessions [51,52]. If we take into account the duration of the sessions, the time would range from a minimum of 45 min [11,47,52,53,54] to a maximum of 90 [55]. Our program would, therefore, be in this range of duration, coinciding with studies that last for 5 weeks [46,50] and carry out 45-min sessions [11,47,53,54]. However, the frequency of the sessions of our intervention does not coincide with the mentioned studies, as we have applied two sessions per week. Nevertheless, this frequency would fall within the frequencies that are being applied in other studies which are between one and five sessions per week [28].

The type of treatment approach used in cognitive interventions has been widely discussed in the literature consulted. We decided to apply a group intervention in our study, which has been supported by different scientific studies [55,56,57,58,59]. In particular, Ermini-Füngschilling et al. [60], advocated for group intervention in mild stages of dementia as it improves social competence. We also agree with the authors in carrying out structured sessions appropriate to the individual needs of the patients despite being in a group. Group stimulation has been shown to be beneficial for people with cognitive impairment, not only affecting cognition but also helping to foster socialization, improve communication, and increase awareness about the disorder [7].

The areas treated (spatial-temporal orientation, memory, attention and concentration, executive function, verbal and written language, praxis, gnosis, association, and categorization) coincide with several well-known programs in our country, such as the Comprehensive Psychostimulation Programme [61] and the Comprehensive Cognitive Action Programme [18]. In this regard, our study is also in line with recent research on the cognitive approach to dementia [11,62]. We coincide with them in the methodology followed: a group of patients with a homogeneity of impaired and preserved abilities as far as possible, two professionals for each session, same room and same arrangement of patients in the room, maintenance of the same timetables, starting and ending the session with a verbal act and groups of patients between five and ten at most [18,61,62,63,64]. Our sample size (n = 58) is also consistent (although slightly higher) with other studies in which the sample ranges from n = 27 to n = 56 [47,49,54,65,66].

Even though cognitive stimulation programs can be administered by any person with previous training and experience, we chose the figure of the occupational therapist to conduct our treatments. This coincides with the authors Yuill and Hollis [67] who conclude that in people with mild to moderate dementia, these health professionals are especially suitable for carrying out cognitive stimulation due to the knowledge that they have and the fact that their approach is oriented towards functionality and recovery of skills. In this respect, our program differs from those applied by other professionals and where cognitive functions have been stimulated with activities related to daily living. We have not focused on stimulation with standard exercises or exercises related to the cognitive function itself, but all activities were related to everyday aspects. This is a novel approach and it is based on the need to generalize the benefits of stimulation to the everyday context of the patients [7,19,28,29] and to use daily activities as an intervention approach [68,69,70]. All these similarities support the methodological basis of our program, and therefore help us to give consistency to our results.

Some of the limitations of the study have been mentioned throughout this section. However, we consider that another limitation of this pilot study was the number of losses which were not factored into the predetermined sample size of 25 in each treatment group. The main reason for the drop-outs of the participants was their transfer to other residential homes. This was external and unrelated to the study itself, and therefore, it was difficult to control. We consider that these losses could have influenced the results in a negative way, not showing all of the improvements that our treatment program could achieve. However, the drop-outs occurred in the follow-up phase and not during the intervention (5 weeks). In addition, the fact that not all participants in the sample had the same type of dementia may be another limitation of the study; although this may make access to larger samples more difficult.

## 5. Clinical Implications

The results of the present study can have important ramifications in the clinical practice. The data show that our cognitive stimulation occupational therapy program maintains cognitive level and improves some cognitive functions in older adults with dementia. Cognitive stimulation is increasingly used nowadays by occupational therapists and other health professionals who can perform this therapy safely if the appropriate training has been completed. In addition, it is a treatment that can be applied with minimal costs.

## 6. Conclusions

The results of the present study provide the preliminary evidence of the feasibility of the cognitive training occupational therapy program. This program showed positive effects on the maintenance of the global cognitive state of institutionalized older adults with dementia and improved significantly the comprehension of verbal commands and praxis.

## Figures and Tables

**Table 1:** Socio-demographic characteristics of the sample at baseline.

Demographics/ Clinical Data		Intervention Group		Control Group		*p*
	Category	N	%	N	%	
Gender	Male	8	28.6	5	16.7	0.277 ^a^
	Female	20	71.4	25	83.3	
Education level (N = 57)	No studies	2	7.4	2	6.7	0.094 ^b^
	Primary School	22	81.5	28	93.3	
	Secondary School	3	11.1	0	0.0	
Date of institutionalization	−2008	5	17.9	4	13.3	0.180 ^b^
	2009–2010	3	10.7	9	30.0	
	2011–2012	20	71.4	17	56.7	
Type of dementia	Alzheimer’s	4	14.3	8	2.7	0.127 ^b^
	Vascular	6	21.4	8	26.7	
	Mixed	1	3.6	2	6.7	
	Lewy body	0	0.0	2	6.7	
	Frontotemporal	0	0.0	1	3.3	
	Not specified	17	60.7	9	30.0	
Neuroleptic treatment (N = 54)	Yes	10	35.7	5	19.2	0.177 ^a^
	No	18	64.3	21	80.8	
Anxiolytic treatment (N = 54)	Yes	10	35.7	5	19.2	0.177 ^a^
	No	18	64.3	21	80.8	
Antidepressive treatment (N = 54)	Yes	6	21.4	12	46.2	0.054 ^a^
	No	22	78.6	14	53.8	
Analgesic treatment (N = 54)	Yes	0	0.0	1	3.8	0.223 ^b^
	No	28	100	25	96.2	
Other treatments of associated pathologies (N = 54)	Yes	8	28.6	5	19.2	0.422 ^a^
	No	20	71.4	21	80.8	
Occupational therapy treatment—cognitive program	Yes	16	57.1	21	70.0	0.309 ^a^
	No	12	42.9	9	30.0	
Occupational therapy treatment—functional rehabilitation program	Yes	11	39.3	13	43.3	0.754 ^a^
	No	17	60.7	17	56.7	
Occupational therapy treatment—ADL	Yes	28	100	30	100	*
	No	0	0.0	0	0.0	
Occupational therapy treatment—leisure	Yes	22	78.6	28	93.3	0.098 ^b^
	No	6	21.4	2	6.7	
Occupational therapy treatment—psychomotricity	Yes	5	17.9	7	23.3	0.607 ^a^
	No	23	82.1	23	76.7	
**Demographics/Clinical Data**		**Intervention Group**		**Control Group**		***p***
		**Media**	**SD**	**Media**	**SD**	
Age		84.21	7.781	81.87	6.673	0.222 ^c^
Diagnosis of dementia (years)		3.18	2.294	4.03	3.409	0.307 ^d^

* The variable is continuous; ^a^ Pearson’s χ^2^; ^b^ ratio of verisimilitude. ^c^ *t*-Student test, equal variances; ^d^ U of Mann–Whitney test. In the Kolmogorov–Smirnov test, the variable cognitive level showed normality (*p*-values superior to 0.05). Note: SD: standard deviation, *p*: *p*-value, ADL: activities of daily living.

**Table 2:** Normality Tests.

	Global				Intervention Group				Control Group			
Outcome Measure	Mean	SD	Z	*p* *	Mean	SD	Z	*p* *	Mean	SD	Z	*p* *
Initial LCMT Test	21.40	3.287	1.153	0.140	21.14	3.363	0.893	0.403	21.63	3.253	1.038	0.232
Week 5 LCMT Test	22.64	3.582	0.948	0.329	23.11	3.833	0.785	0.569	22.20	3.336	0.769	0.595
Week 12 LCMT	21.64	3.970	0.845	0.473	21.85	4.475	0.835	0.489	21.42	3.469	0.808	0.532

*p* *: *p*-value for the Kolmogorov–Smirnov test to check normality, SD: standard deviation; Z: Z of Kolmogorov–Smirnov; LCMT: Lobo’s Cognitive Mini Test.

**Table 3:** Cognitive level (Lobo’s Cognitive Mini Test) of the patients after the application of the treatments.

Outcome Measure	Intervention Group		Control Group		*p*
	Mean	SD	Media	DT	
LCMT at 0 weeks	21.14	3.363	21.63	3.253	0.574 ^a^
LCMT at 5 weeks	23.11	3.833	22.20	3.336	0.340 ^a^
LCTM at 12 weeks	21.85	4.475	21.42	3.469	0.741 ^a^

^a^ T-Student test. Equal variances. SD: standard deviation. LCMT: Lobo’s Cognitive Mini Test.

**Table 4:** Comparison between the control and the intervention group of the cognitive level after the intervention according to the severity of dementia.

GDS at 5 Weeks	Control Group			Intervention Group		
	Mean	SD	*p*	Mean	SD	*p*
No cognitive decline	*	*	<0.001 ^a^	30.00	0.000	<0.001 ^a^
Very mild cognitive decline	25.43	4.077		27.17	1.835	
Mild cognitive decline	22.44	1.504		22.64	2.560	
Moderate cognitive decline (mild dementia)	18.43	1.618		19.57	3.101	

* There are no controls with no cognitive impairment. ^a^ ANOVA. SD: standard deviation.

## Data Availability

The data underlying this article cannot be shared publicly to maintain the privacy of individuals that participated in the study. The data will be shared on reasonable request to the corresponding author.

## Supplementary Materials

The following supporting information can be downloaded at: https://www.mdpi.com/article/10.3390/jpm12111808/s1. Table S1: Organizational chart of the treatment program, Table S2: Example of a session within the occupational therapy ADL cognitive stimulation program.

## References

[1] Asociación Americana de Psiquiatría (2014). DSM-5 Manual Diagnóstico y Estadístico de Los. Trastornos Mentales.

[2] Custodio N., Montesinos R., Alarcón J.O. (2018). Evolución histórica del concepto y criterios actuales para el diagnóstico de demencia. Rev. Neuropsiquiatr..

[3] Alzheimer’s Disease International (2015). The Global Impact of Dementia. An analysis of prevalence, Incidence, Cost and Trends. World Alzheimer Report 2015.

[4] Galende A.V., Ortiz M.E., Velasco S.L., Luque M.L., Miguel C.L.D.S.D., Jurczynska C.P. (2021). Informe de la Fundación del Cerebro. Impacto social de la enfermedad de Alzheimer y otras demencias. Neurología.

[5] Ramos P., Serrano P., Ribera J.M., Bermejo F., Vega S., Gil P. (2007). La Enfermedad de Alzheimer y Otras Demencias Detección y Cuidados en las Personas Mayores. Madrid. https://fiapam.org/wp-content/uploads/2013/12/235-comunidadmadrid-enfermedad-011.pdf.

[6] Clare L., Woods R.T., Moniz E.D., Orrell M., Spector A. (2008). Rehabilitación Cognitive y Entrenamientos Cognitive Para la Enfermedad de Alzheimer y la Demencia Vascular de Estadío Temprano. Cochrane Database of Systematic Reviews Review. https://www.cochranelibrary.com/cdsr/doi/10.1002/14651858.CD003260/full/es.

[7] Cordero P.R., Yubero R. (2016). Tratamiento no farmacologico del deterioro cognitivo. Rev. Esp. Geriatr. Gerontol..

[8] Lorenzo J., Fontan L. (2001). La rehabilitación de los trastornos congitivos. Rev. Med. Urug..

[9] Gay F.J., González V., Pablos C. (2011). Guía de Orientación en la Práctica Profesional de la Valoración Reglamentaria de la Situación de Dependencia en Personas con Enfermedad de Alzheimer y otras Demencias. https://fiapam.org/wpcontent/uploads/2012/10/GuiaOrientacion.pdf.

[10] Zamarrón M.D., Tárraga L., Fernández R. (2008). Plasticidad cognitiva en personas con la enfermedad de Alzheimer que reciben programas de estimulación cognitiva. Psicothema.

[11] Gómez I., Andrés E.M., Gómez A., Peralta P. (2021). Análisis del efecto a largo plazo de un programa de estimulación cognitiva en mayores con deterioro cognitivo leve en atención primaria: Ensayo controlado aleatorizado. Atención Primaria.

[12] Clare L., Woods R.T. (2004). Cognitive training and cognitive rehabilitation for people with early-stage Alzheimer‘s disease: A review. Neuropsychol. Rehabil..

[13] Franco M.A., Orihuela T., Buenos Y., Cid T. (2000). Programa Gradior. Programa de Evaluación y Rehabilitación Cognitiva Por Ordenador.

[14] Martínez T. (2002). Estimulación Cognitiva: Guía y Material para la Intervención. Asturias (España): Gobierno del Principado de Asturias.

[15] Montejo P., Montenegro M., Reinoso A.I., De Andrés M.E., Claver M.D. (1997). Programa de Memoria.

[16] Tárraga L., Fernández Ballesteros R., Nicolás J. (1991). Programa de Psicoestimulación Integral (PPI). Tratamientos de psicoestimulación. Libro Blanco Sobre la Enfermedad de Alzheimer.

[17] Tárraga L., Boada M., Morera A., Domènech S., Llorente A. (1999). Volver a empezar. Ejercicios Prácticos de Estimulación Cognitiva Para Enfermos de Alzheimer.

[18] García J., Carro J. (2011). Programa de Actuación Cognitiva Integral en Demencia.

[19] Matilla-Mora R., Martínez-Piédrola R.M., Huete J.F. (2016). Eficacia de la terapia ocupacional y otras terapias no farmacológicas en el deterioro cognitivo y la enfermedad de Alzheimer. Rev. Española Geriatría Gerontol..

[20] Tarraga L., Fernández-Ballesteros R., Díez Nicolás J. (2001). Tratamiento de psicoestimulación. Libro Blanco Sobre la Enfermedad de Alzheimer y Trastornos Afines.

[21] Peña-Casanova J. (1999). Activemos la Mente.

[22] Associations of Relatives of Alzheimer’s and other Dementia Patients (Asociaciones de Familiares de Enfermos de Alzheimer y otras Demencias—AFAL) (2003). El Baúl de los Recuerdos.

[23] Fernández R., Zamarrón M.D., Tárraga L., Moya R., Iñiguez J. (2003). Cognitive Plasticity in Healthy, Mild Congitive Impairment (MCI) Subjects and Alzheimer‘s Disease Patients: A Research Project in Spain. Eur. Psychol..

[24] Santos del Riego S. (2005). El ser humano como ser ocupacional. Rehabilitación.

[25] Salmon N. (2006). Cognitive stimulation therapy versus acetyl cholinesterase inhibitors for mild to moderate dementia: A latter-day David and Goliath?. Br. J. Occup. Ther..

[26] American Occupational Therapy Association (2014). Occupational therapy practice framework: Domain and process. Am. Occup. Ther. Assoc..

[27] Francés I., Barandiarán M., Marcellán T., Moreno L. (2003). Estimulacion psiconognitiva en demencia. Anales. Sis. San. Navar..

[28] Bahar-Fuchs A., Martyr A., Goh A.M., Sabates J., Clare L. (2019). Entrenamiento cognitivo para personas con demencia leve a moderada. Cochrane Database Syst. Rev..

[29] Woods R., Aguirre E., Spector A.E., Orrell M. (2012). Cognitive stimulation to improve cognitive functioning in people with dementia. Cochrane Database Syst. Rev..

[30] Lucinda Aguirre-Cruz M., Cruz Aguilera D.L., San Juan Orta D. (2016). Líneas de investigación en demencias en el Instituto Nacional de Neurología y Neurocirugía Manuel Velasco Suárez. Arch. Neurocien. (Mex.).

[31] Moher D., Hopewell S., Schulz K.F., Montori V., Gøtzsche P.C., Devereaux P.J., Elbourne D., Egger M., Altman D.G. (2010). CONSORT 2010 explanation and elaboration: Updated guidelines for reporting parallel group randomised trials. J. Clin. Epidemiol..

[32] Boutron I., Moher D., Altman D.G., Schulz K.F., Ravaud P., CONSORT Group (2008). Extending the CONSORT Statement to Randomized Trials of Nonpharmacologic Treatment: Explanation and Elaboration. Ann. Intern. Med..

[33] American Psychiatric Association (2000). Diagnostic and Statistical Manual of Mental Disorders.

[34] Solís Barrero C.L., García Arrioja S., Ojeda Manzano A. (2005). Indice de Barthel (IB): Un instrumento esencial para la evaluación funcional y la rehabilitación. Plast Restauración Neurológica.

[35] Lobo A., Sanz O., Marcos G. (1999). Revalidación y normalización del mini examen cognoscitivo (primer versión en castellano del Mini Mental Status Examination) en la población general geriátrica. Med. Clin..

[36] Fernandez E.J., Sanchez C., Monroy M.L., Barbero F.J., Calvo J.I. (2018). Estudio aleatorizado de un programa de entrenamiento de cognición cotidiana frente a estimulación cognitiva tradicional en adultos mayores. Gerokomos.

[37] Tárraga L. (1994). Estrategia no Farmacológica del Deterioro Cerebral Senil y Demencia. Medicine.

[38] Reisberg B., Ferris S.H., De Leon M.J., Crook T. (1982). The Global Deterioration Scale for assessment of primary degenerative dementia. Am. J. Psychiatry.

[39] Broche Y. (2017). Instrumental alternatives for brief cognitive screening of the elderly: Beyond the Minimental Test. Rev. Cuba. Med. Gen. Integr..

[40] Lee C.T. (2003). Introductory Biostatistics.

[41] Orgeta V., McDonald K.R., Poliakoff E., Hindle J.V., Clare L., Leroi I. (2020). Cognitive training interventions for dementia and mild cognitive impairment in Parkinson’s disease. Cochrane Database Syst. Rev..

[42] Chandra S.R., Issac T.G., Abbas M.M. (2015). Apraxias in Neurodegenerative Dementias. Indian J. Psychol. Med..

[43] Olazarán J., Reisberg B., Clare L., Cruz I., Peña-Casanova J., Del Ser T., Woods B., Beck C., Auer S., Lai C. (2010). Nonpharmacological Therapies in Alzheimer’s Disease: A Systematic Review of Efficacy. Dement. Geriatr. Cogn. Disord..

[44] Villalba S., Espert R. (2014). Estimulación cognitiva: Una revisión neuropsicológica. Therapeia.

[45] Rubinstein W., Politis D. (2006). Estudio sobre la apraxia y las actividades de la vida diaria en relación al grado de severidad de la demencia. XIII Jornadas de Investigación y Segundo Encuentro de Investigadores en Psicología del Mercosur.

[46] Breuil V., De Rotrou J., Forette F., Tortrat D., Ganansia-Ganem A., Frambourt A., Moulin F., Boller F. (1994). Cognitive stimulation of patients with dementia: Preliminary results. Int. J. Geriatr. Psychiatry.

[47] Spector A., Orrell M., Davies S., Woods R. (2001). Can reality orientation be rehabilitated? Development and piloting of an evidence-based programme of cognition-based therapies for people with dementia. Neuropsychol. Rehabil..

[48] Bach D., Bach M., Böhmer F., Frühwald T., Grilc B. (1995). Reactivating Occupational Therapy: A Method to improve Cognitive Performance in Geriatric Patients. Age Ageing.

[49] Matsuda O. (2006). Cognitive stimulation therapy for Alzheimer’s disease: The effect of cognitive stimulation therapy on the progression of mild Alzheimer’s disease in patients treated with donepezil. Int. Psychogeriatr..

[50] Chapman S.B., Weiner M.F., Rackley A., Hynan L.S., Zientz J. (2004). Effects of Cognitive-Communication Stimulation for Alzheimer’s Disease Patients Treated with Donepezil. J. Speech Lang. Hear. Res..

[51] Koh K., Ray R., Lee J., Nair A., Ho T., Ang P.C. (1994). Dementia in Elderly Patients: Can the 3R Mental Stimulation Programme improve Mental Status?. Age Ageing.

[52] Spector A., Thorgrimsen L., Woods R., Royan L., Davies S., Butterworth M., Orrell M. (2003). Efficacy of an evidence-based cognitive stimulation therapy programme for people with dementia. Br. J. Psychiatry.

[53] Woods R.T., Thorgrimsen L., Spector A., Royan L., Orrell M. (2006). Improved quality of live and cognitive stimulation in dementia. Aging Ment. Health.

[54] Orrell M., Spector A., Thorgrimsen L., Woods B. (2005). A pilot study examining the effectiveness of maintenance cogni-tive stimulation therapy (MCST) for people with dementia. Int. J. Geriatr. Psychiatry.

[55] Ermini D., Meier D. (1995). Memory training: An important part of a “milieu therapy” for patients with senile dementia. Z. Fuer Gerontol. Und Geriatr..

[56] Moore S., Sandman C.A., McGrady K., Kesslak J.P. (2001). Memory training improves cognitive ability in patients with dementia. Neuropsychol. Rehabil..

[57] Koltai D.C., Welsh-Bohmer K.A., Smechel D.E. (2001). Influence of anosognosia on treatment outcome among dementia patients. Neuropsychol. Rehabil..

[58] Kesslak J., Nackoul K., Sandman C. (1997). Memory training for individuals with Alzheimer’s disease improves name recall. Behav. Neurol..

[59] Bernhardt T., Maurer K., Frölich L. (2002). Influence of a memory training program on attention and memory performance of patients with dementia. Z. Fuer Gerontol. Und Geriatr..

[60] Ermini D., Hendriksen C., Meier D., Regard M., Stähelin H., Fitten J., Frisoni G., Vellas B. (1998). Entrenamiento cognitivo en pacientes externos con demencia leve: Efectos sobre el estado de ánimo y las funciones cognitivas. Investigación y Práctica en la Enfermedad de Alzheimer.

[61] Tárraga L. (1998). Terapias blandas: Programa de Psicoestimulacion Integral. Alternativa terapéutica para las personas con enfermedad de Alzheimer. Rev. Neurol..

[62] Gomez-Soria I., Peralta-Marrupe P., Plo F. (2020). Cognitive stimulation program in mild cognitive impairment A randomized controlled trial. Dement. Neuropsychol..

[63] Arroyo E.M. (2002). Estimulación Psicocognoscitiva en las Demencias.

[64] Grupo de trabajo de la Guía de Práctica Clínica sobre la atención integral a las personas con enfermedad de Alzheimer y otras demencias (2010). Guía de Práctica Clínica sobre la atención integral a las personas con enfermedad de Alzheimer y otras demencias. Plan de Calidad para el Sistema Nacional de Salud del Ministerio de Sanidad, Política Social e Igualdad.

[65] Buschert V.C., Friese U., Teipel S.J., Schneider P., Merensky W., Rujescu D., Möller H.-J., Hampel H., Buerger K. (2011). Effects of a Newly Developed Cognitive Intervention in Amnestic Mild Cognitive Impairment and mild Alzheimer’s disease: A Pilot Study. J. Alzheimer’s Dis..

[66] Coen R.F., Flynn B., Rigney E., O’Connor E., Fitzgerald L., Murray C., Dunleavy C., McDonald M., Delaney D., Merriman N. (2011). Efficacy of a cognitive stimulation therapy programme for people with dementia. Ir. J. Psychol. Med..

[67] Yuill N., Hollis V. (2011). A Systematic Review of Cognitive Stimulation Therapy for Older Adults with Mild to Moderate Dementia: An Occupational Therapy Perspective. Occup. Ther. Int..

[68] Schell B.A.B., Gillen G. (2016). Scaffa ESC. Terapia Ocupacional.

[69] Roley S.S., DeLany J.V., Barrows C.J., Brownrigg S., Honaker D., Sava D.I., Talley V., Voelkerding K., Amini D.A., Smith E. (2008). Occupational Therapy Practice Framework: Domain & Process 2nd Edition. Am. J. Occup. Ther..

[70] Ciro C.A., Anderson M.P., Hershey L.A., Prodan C.I., Holm M.B. (2015). Instrumental Activities of Daily Living Performance and Role Satisfaction in People with and Without Mild Cognitive Impairment: A Pilot Project. Am. J. Occup. Ther..

