# Supplementary material for: Effects of a Cognitive Stimulation Program in Institutionalized Patients with Dementia
Source: J Pers Med. 2022 Nov 1;12(11):1808. doi: 10.3390/jpm12111808 (PMC9698764; doi:10.3390/jpm12111808)
Supplement: Supplementary file 1 [file jpm-12-01808-s001.zip › jpm-1929842-supplementary.pdf]

Table S1. Organisational chart of the treatment programme.

Week	Session 1	Session 2
**First**	Temporal-spatial orientation<br>Attention /concentration<br>Gnosia	Temporal-spatial orientation<br>Attention /concentration<br>Praxis
**Second**	Temporal-spatial orientation<br>Attention /concentration<br>Executive function (planification)	Temporal-spatial orientation<br>Attention /concentration<br>Semantic memory
**Third**	Temporal-spatial orientation<br>Attention /concentration<br>Sequencing	Temporal-spatial orientation<br>Attention /concentration<br>Categorisation
**Fourth**	Temporal-spatial orientation<br>Attention /concentration<br>Language/verbal fluency	Temporal-spatial orientation<br>Attention /concentration<br>Visual discrimination
**Fifth**	Temporal-spatial orientation<br>Attention /concentration<br>Dressing praxis	Temporal-spatial orientation<br>Attention /concentration<br>Executive function

Table S2: Example of a session within the Occupational Therapy ADL cognitive stimulation programme

1.

Cognitive function	Objective of the activity	Activity
**Temporal-spatial orientation**	To improve the orientation in time and space	With a picture of a clock, the patient has to indicate:<br>- what time it is,<br>- which ADL he/she performs at that time of the day,<br>- where does he/she perform that ADL.
**Attention**	To stimulate the sustained attention through recognition	With a worksheet with different types of trousers, dresses and underwear, the patient has to indicate those that have the same colour.
**Categorisation**	To train the recognition of sematic categories and to improve categorisation in activities of daily living	With a worksheet that presents the picture of a table, the patient must write 5 types of food that need to be eaten with a spoon and 5 that need to be eaten with a fork.<br>Then, different categories and words that correspond to those categories are presented to the patient. He/she must tick the word that does not correspond to the category indicated by the therapist.
